# Effect of Crystal Orientation on Femtosecond Laser-Induced Thermomechanical Responses and Spallation Behaviors of Copper Films

**DOI:** 10.1038/s41598-017-09559-6

**Published:** 2017-08-23

**Authors:** Qi-lin Xiong, Zhenhuan Li, Takayuki Kitamura

**Affiliations:** 1 Department of Mechanics, Huazhong University of Science & Technology, 1037 Luoyu Road, Wuhan, 430074 China; 2 Hubei Key Laboratory of Engineering Structural Analysis and Safety Assessment, Luoyu Road 1037, Wuhan, 430074 China; 3 Department of Mechanical Engineering and Science, Kyoto University, Nishikyo-ku, Kyoto, 615-8540 Japan

## Abstract

Ultrafast thermomechanical responses and spallation behaviours of monocrystal copper films irradiated by femtosecond laser pulse are investigated using molecular dynamics simulation (MDS). Films with 〈100〉, 〈110〉 and 〈111〉 crystal orientations along the thickness direction were studied. The results show that the crystal orientation has a significant effect on femtosecond laser-induced thermomechanical responses and spallation behaviors of monocrystal copper films. The discrepancy between normal stresses in copper films with different crystal orientation leads to distinct differences in lattice temperature. Moreover, the copper films with different crystal orientations present distinct spallation behaviors, including structural melting (atomic splashing) and fracture. The melting depth of 〈100〉 copper film is lower than that of 〈110〉 and 〈111〉 copper films for the same laser intensity. The dislocations and slip bands are formed and propagate from the solid-liquid interface of 〈110〉 and 〈111〉 copper films, while these phenomena do not appear in 〈100〉 copper film. Additionally, numerous slip bands are generated in the non-irradiated surface region of copper films due to reflection of mechanical stress. These slip bands can finally evolve into cracks (nanovoids) with time, which further result in the fracture of the entire films.

## Introduction

Femtosecond laser processing of metal is attracting increasing attention of researchers due to its high machining efficiency and high precision machining on metal^1–4^. To achieve high precision machining of metal, a profound understanding of thermomechanical interaction between femtosecond laser and metal is indispensable. A great deal of research on femtosecond laser-metal interaction has been carried out in the past years. Due to extremely high temperatures and strain rates induced by femtosecond laser pulse, the thermophysical properties of materials, which usually measured experimentally at room temperature, are out of the reach of continuum mechanics^5^ in such extreme situations. However, these extreme situations can be simulated by molecular dynamics simulation (MDS) because MDS can take into account the temperature and strain rate-dependent thermophysical properties of materials. Under these circumstances MDS has been widely adopted to study the ultrafast thermomechanical interaction in the metal induced by ultrashort laser. For instance, Huang and Lai^6^ studied femtosecond laser induced nucleation and propagation of dislocations in the copper film with 〈100〉 crystal orientation along thickness direction using MDS. Gan and Chen^7^ investigated the ultrafast nonthermal ablation of gold nanofilms with 〈100〉 crystal orientation along thickness direction using MDS. They found that the film thickness has a significant influence on the evolution process of the stress wave. Xiong *et al*.^8, 9^ used MD simulation to investigate the electron relaxation effect on femtosecond laser-induced thermoelastic response of gold films with 〈100〉 crystal orientation along thickness direction and the effect of femtosecond laser trains on ultrafast thermomechanical responses of a 〈100〉 copper film. Their results illustrated that the electron relaxation effect can be neglected when the laser duration is much longer than the electron thermal relaxation time; but it becomes significant if the laser duration matches the electron relaxation time, especially when the former is much shorter than the latter.

Since the interactions between atoms are strongly affected by the distance between atoms in a single crystal material, the material parameter of the single crystal material varies with the crystal orientation^10, 11^. Thus, it is very important to find out how the crystal orientation influences the ultrafast thermomechanical responses and spallation behaviors of material for application of femtosecond laser processing in engineering. Unfortunately, only the single crystal metal film with 〈100〉 crystal orientation along thickness direction is used to investigate femtosecond laser-metal interaction in the existing studies^6–9^. Few works about the influence of crystal orientation on ultrafast thermomechanical responses and spallation behaviors of single crystal metal during femtosecond laser heating has been reported up to now.

The main aim of the present work is to study the effect of crystal orientation on ultrafast thermomechanical responses and spallation behaviors of monocrystal copper films irradiated by femtosecond laser pulse. Films with thickness orientations along the typical 〈100〉, 〈110〉 and 〈111〉 crystallographic directions are simulated using MDS. The results reveal that the crystal orientation has a significant effect on femtosecond laser-induced thermomechanical responses of copper films. Due to thermo-mechanical coupling interaction, the discrepancy between normal stresses in copper films with different crystal orientation leads to distinct differences in lattice temperature. The spallation behaviors, including structural melting (atomic splashing), generation of void and fracture of copper films, are different for films with different crystal orientations. Specifically, the melting part of 〈100〉 copper film will not fly away from the shallow surface region at 0.2 ns, while for its 〈110〉 and 〈111〉 counterparts the melting parts can fly away from the surface at about 0.15 ns, which indicates that, compared with 〈110〉 and 〈111〉 copper films, it is more difficult to ablate 〈100〉 copper film. The size of voids generated in melting subsurface of 〈110〉 and 〈111〉 copper films is larger than that in 〈100〉 copper film. The dislocation and slip band are formed and propagate from the solid-liquid interface for 〈110〉 and 〈111〉 copper films. In addition, lots of slip bands are generated in the non-irradiated surface region of copper films due to mechanical stress. The slip bands in 〈100〉 copper film disappear when stress wave passes; however, these slip bands are developed into cracks (nanovoids), which finally result in fracture of copper films.

## Process of MD Simulation

**Figure 1:**
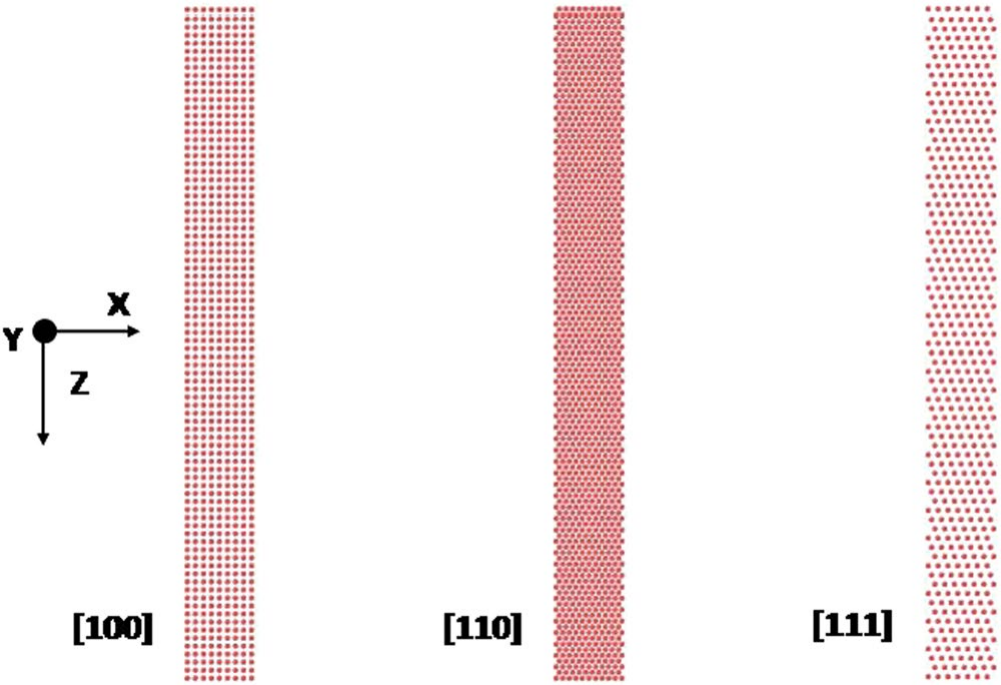
Atomic models of copper films with 〈100〉, 〈110〉 and 〈111〉 crystal orientation along thickness direction.

To study the effect of crystal orientation on ultrafast thermo-mechanical coupling responses and spallation behaviors of monocrystal copper films, three MD models of thin copper films are created: i) a monocrystal copper target with the 〈1 0 0〉, 〈0 1 0〉 and 〈0 0 1〉 crystal orientations along the x-, y- and z-directions, respectively, which has a size of 15.2 nm × 15.2 nm × 455.5 nm and possesses 8.68 million atoms as shown in Fig. (1); ii) a monocrystal copper target with the 〈1–1 1〉, 〈1–1 2〉 and 〈1 1 0〉 crystal orientations along the x-, y- and z-directions, respectively, whose size is 15.0 nm × 15.0 nm × 451.6 nm; iii) a monocrystal copper target with the 〈−1 1 0〉, 〈−1–1 2〉 and 〈1 1 1〉 crystal orientations along the x-, y- and z-directions, respectively, whose size is 15.0 nm × 15.0 nm × 451.6 nm. According to the previous studies^12^, the cross-sectional size of 15 nm × 15 nm size is adopted to avoid significant effects of periodic boundaries on the dislocation nucleation dynamics. In addition, for the 〈1 1 0〉 and 〈1 1 1〉 copper films, the cross-sectional sizes (x-y plane) are slightly adjusted in order to achieve the perfect periodic boundary conditions.

The ultrafast thermo-mechanical interaction between femtosecond laser and thin copper film is investigated using the combined hyperbolic two-temperature theory (HTTM)^13^ and MD model^14^ in the present work. The HTTM-MD model is described as follows:

HTTM:

1 $${C}_{e}({T}_{e})\frac{\partial {T}_{e}}{\partial t}=-\frac{\partial {q}_{e}}{\partial z}-G({T}_{e},{T}_{l})({T}_{e}-{T}_{l})+Q(z,t)$$

2 $${\tau }_{e}({T}_{e},{T}_{l})\frac{\partial {q}_{e}}{\partial t}+{q}_{e}=-{k}_{e}({T}_{e},{T}_{l})\frac{\partial {T}_{e}}{\partial z}$$

Here $Q(z,t)$ is the Gaussian laser heat source, which describes the laser-material interaction by the following distribution of femtosecond laser energy along z direction:

3 $$Q(z,t)=\sqrt{\frac{\beta }{\pi }}\frac{{J}_{0}(1-R)}{{t}_{p}{z}_{s}}\exp [-(\frac{z}{{z}_{s}})-\beta {(\frac{t}{{t}_{p}}-2)}^{2}]$$

Here *R* is the reflectivity, *J*_0_ is the peak intensity, *J*_0_(1*-R*) is the absorbed laser fluence, *t*_*p*_ is the laser duration and *z*_*s*_ is the optical penetration depth of copper. In the present study, *t*_*p*_ = 100 fs. The simulation of the femtosecond laser-material interaction starts at *t* = 0, the peak laser energy injection is located at *t* = 2*t*_*p*_. *β* = 4 ln 2, and *z*_*s*_ = 14 nm for the laser wavelength between 10 mm and 250 nm is used in the simulations^15^.

MD:

4 $${m}_{i}\frac{{d}^{2}{r}_{i}}{d{t}^{2}}={F}_{i}({U}_{ij})+\zeta {m}_{i}{v}_{i}^{T}$$

where the coupling coefficient in the Newton equation is given by

5 $$\zeta =\frac{1/n{\sum }_{k=1}^{n}G{V}_{N}({T}_{e}^{k}-{T}_{l})}{\sum _{i}{m}_{i}{({v}_{i}^{T})}^{2}}$$

Here ${v}_{i}^{T}$ denotes the velocity of atom *i* excluding the velocity of mass center of the cell (or thermal velocity); ${F}_{i}({U}_{ij})$ is the forces exerted on atom *i* due to the interaction between atoms; ${V}_{N}$ is the volume of the cell; ${T}_{e}^{k}$ is the average electron temperature at the kth time step of finite difference (FD); ${\sum }_{i}$ is the number of atoms in the volume ${V}_{N}$.

Large-Scale Atomic Molecular Massively Parallel Simulator (LAMMPS)^16^ is used to perform all MD simulations; the OVITO^17^ is used to realize the visualization of atomic structure. In MD simulations, periodic boundary condition is applied in the in-plane direction of film (i.e., x–y plane) and the free boundary condition is assigned to the thickness direction (i.e., z-direction). The simulation technique of the HTTM-MD model described in the existing study^8, 9^ is employed in the present work. The thermo physical parameters of copper used in MD simulations are taken from the literature^9, 15^.

The embedded atom method (EAM) potential^18^ is adopted in the present MD simulations for the interactions between atoms. The EAM potential energy of atom *i* is described as follows:

6 $${E}_{i}={F}_{\alpha }(\sum _{i\ne j}{\rho }_{\beta }({r}_{ij}))+\frac{1}{2}\sum _{i\ne j}{\varphi }_{\alpha \beta }({r}_{ij})$$

where ${F}_{\alpha }$ is the embedding energy function of the electron density ${\rho }_{\beta }({r}_{ij})$, ${\varphi }_{\alpha \beta }$ is a pair potential function and *α*, *β* denotes the element types of atoms *i* and *j*, respectively. The virial stress^19–21^ is applied in the present study, which is computed by

7 $${\sigma }_{\alpha \beta }=-1/V(\sum _{i}{p}_{\alpha }^{^{\prime} }{p}_{\beta }^{^{\prime} }/{m}^{i}+\sum _{i}\sum _{j > 1}{r}_{\alpha }^{ij}{f}_{\beta }^{ij})$$

where ${p}_{\alpha }^{^{\prime} },\,{p}_{\beta }^{^{\prime} }$ are the momentums of atom *i* in the *α*- and *β*-coordinate directions, ${r}_{\alpha }^{ij}$ is the component of the position vector between atoms *i* and *j* in the α-direction, ${f}_{\beta }^{ij}$ is the component of the force vector on atom *i* due to atom *j* in the *β*-direction, *V* is the volume of current cell.

## Results and Discussion

### Effect of Crystal Orientation on Femtosecond Laser-Induced Thermomechanical Responses

**Figure 2:**
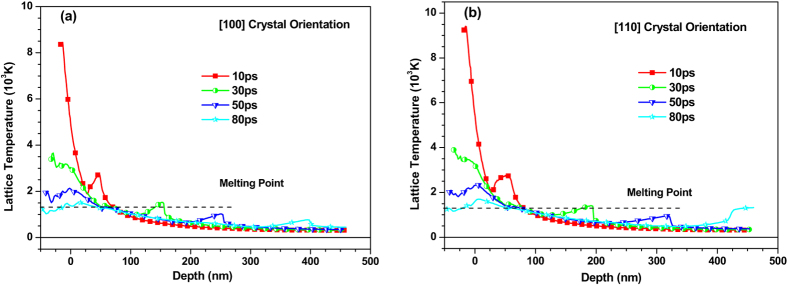
Temperature distribution of (**a**) 〈100〉 and (**b**) 〈110〉 copper films under an absorbed laser intensity of 0.065 J/cm^2^ at 10 ps, 30 ps, 50 ps and 80 ps time instant.

In order to clearly show the contents of figures, only results of 〈100〉 and 〈110〉 copper films are presented in figures. Figure 2 demonstrates temperature distribution in 〈100〉 and 〈110〉 copper films under an absorbed laser intensity of 0.065 J/cm^2^ at the time of 10 ps, 30 ps, 50 ps and 80 ps. Lattice temperature of surface region of 〈100〉 and 〈110〉 copper films decreases gradually with the time as shown in Fig. 2 owing to the fact that the energy of the atomic system is transferred to a deep position through collision interaction between lattices. Additionally, an apparent bump always presents in lattice temperature, whose mechanism has been discussed in the previous studies^8, 9^. Thermomechanical coupling interaction between stress (mechanical field) and temperature (thermal field) leads to this interesting phenomenon.

**Figure 3:**
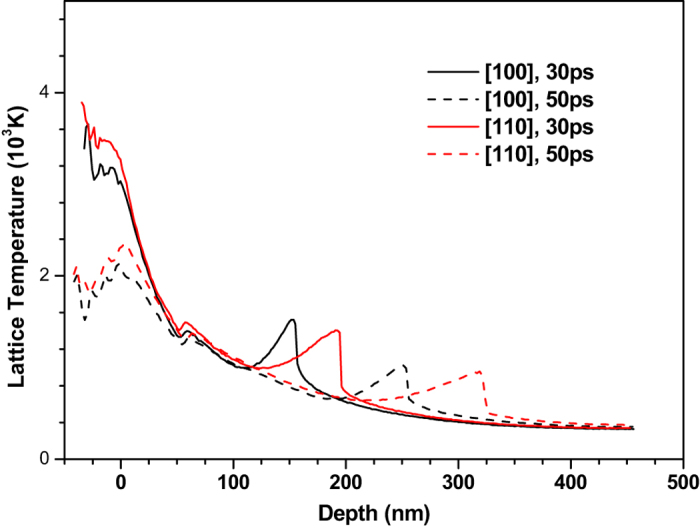
The contrast of temperature distributions of 〈100〉 and 〈110〉 copper films under an absorbed laser intensity of 0.065 J/cm^2^ at 30 ps and 50 ps time instant.

Before 80 ps, the region where the temperature is higher than the melting point (1357 K) of copper extends as time increases. At ~80 ps, the melting depth reaches its maximum value and does not extend to deeper region with the increase of time. The estimated melting depths are about 50 nm and 60 nm for 〈100〉 and 〈110〉 copper films, respectively, which is close to the experimental results^22^. To show the effect of crystal orientation on lattice temperature, the temperature distributions of 〈100〉 and 〈110〉 copper films under an absorbed laser with the same intensity of 0.065 J/cm^2^ at 30 ps and 50 ps is shown in Fig. 3. Lattice temperature in 〈110〉 copper film is found to be slightly higher than that in 〈100〉 copper film. However, the positions of lattice temperature peak show significant difference between 〈100〉 and 〈110〉 copper films, which can be attributed to the fact that the propagation speed of the bump in 〈110〉 copper film is greater than that in the 〈100〉 copper film. The reason leading to different propagation speeds of the bump in 〈100〉 and 〈110〉 copper films will be explained by the following stress analysis.

**Figure 4:**
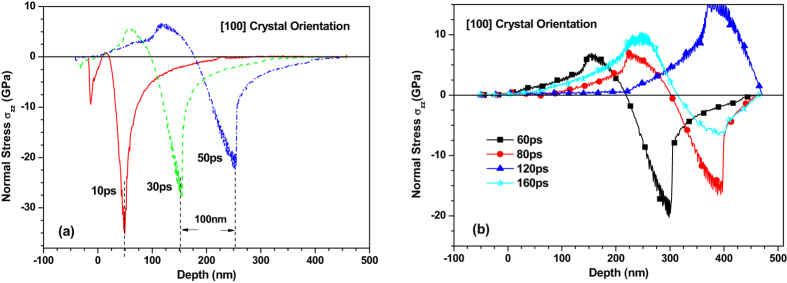
Normal stress (σ_zz_) distribution of 450 nm 〈100〉 copper films under an absorbed laser intensity of 0.065 J/cm^2^ at 10–160ps time instant.

**Figure 5:**
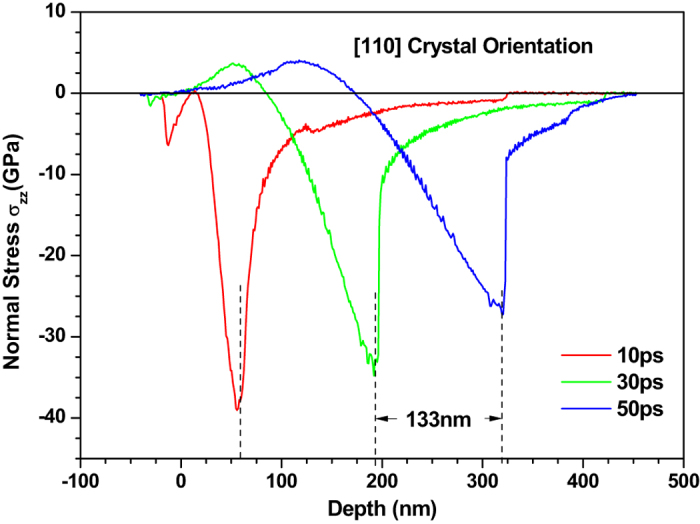
Normal stress (σ_zz_) distribution of 450 nm 〈110〉 copper films under an absorbed laser intensity of 0.065 J/cm^2^ at 10–50 ps time instant.

Figures 4 and 5 depict the normal stress distribution in the 〈100〉 and 〈110〉 copper films under an absorbed laser intensity of 0.065 J/cm^2^ at 10 ps, 30 ps, 50 ps, 60 ps, 80 ps, 120 ps and 160 ps, respectively. At 10 ps, there is a compressive stress formed near the top surface which is irradiated by femtosecond laser. The sharp rise of lattice temperature in the surface region of thin film due to femtosecond laser heating results in seriously unbalanced lattice temperature distribution as shown in Figs 2 and 3. Such a unbalanced lattice temperature distribution further causes ultrafast deformation of thin film and thus limits the deformation inside thin film, which leads to the sharp compressive stress. As time increases, the material near the irradiated surface is stretched due to thermal expansion. Meanwhile, the tensile stress appears in the irradiated surface as shown in Fig. 4. The maximum stress of ~36.0 GPa in the 〈100〉 thin film under the laser intensity of 0.065 J/cm^2^ is slightly lower than that in the 〈110〉 thin film. It is worth noting that the stress wave propagates at an invariable speed. After dividing the time interval into the dimensional interval the propagation speed of the stress wave is estimated to be about 5000 m/s and 6600 m/s for the 〈100〉 and 〈110〉 copper films, respectively.

According to the thermal stress theory of continuum mechanics^23^, the coefficient $\delta$ of coupling between thermal (lattice temperature) and mechanical (deformation) is given as follows:

8 $$\delta \propto \frac{{\alpha }^{{\rm{2}}}E}{\rho {c}_{\sigma }}$$

where

9 $${c}_{\sigma }={c}_{\rho }+\frac{{\alpha }^{2}{T}_{0}E}{\rho }$$

**Figure 6:**
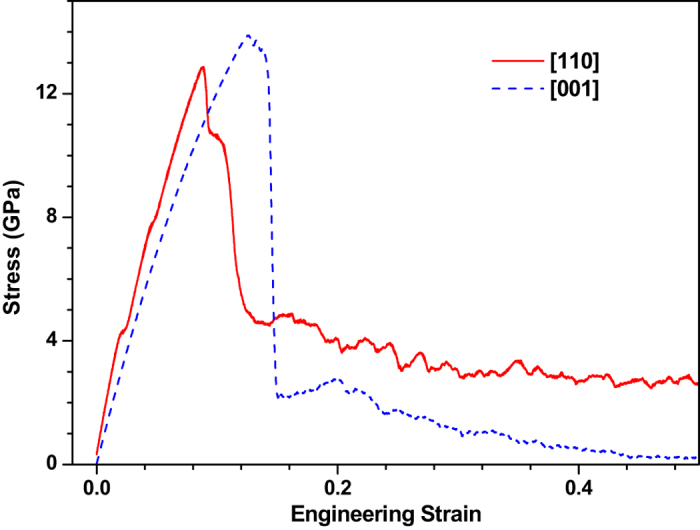
Stress-strain curves of 〈110〉 and 〈100〉 crystal orientations under the tensile strain rate of 10^9^s^−1^ at room temperature.

Here, ${c}_{\rho }$ is specific heat at constant strain, *α* is thermal expansion coefficient, ***E*** is elastic modulus, *ρ* is mass density. The coupling parameter is a function of thermal expansion coefficient, elastic modulus and so on. The thermal expansion coefficient of copper is significantly dependent on the crystal orientation^24^. Specifically, the thermal expansion coefficient of 〈110〉 orientation is greater than that of 〈100〉 orientation. The tensile stress-strain curves for 〈110〉 orientation and 〈100〉 orientation are plotted in Fig. 6. By calculating the slope of the linear part in Fig. 6, we find that the elastic modulus in the 〈110〉 orientation is higher than that in the 〈100〉 orientation, which is consistent with the conclusion of the previous study^25^. Since the mass density and specific heat are assumed to be orientation-independent, thermo-mechanical coupling in the 〈110〉 orientation is stronger than that in the 〈100〉 orientation according to a simple mathematical estimation. This result indicates that under the same laser intensity, the mechanical response (stress) induced in the 〈110〉 orientation is greater than that in the 〈100〉 orientation. The phenomenon shown in Figs 4 and 5 can thus be well explained based on this finding.

According to the theory of continuum mechanics^26^, the elastic wave speed is calculated by

10 $${V}_{e}=\sqrt{\frac{E(1-\upsilon )}{(1+\upsilon )(1-2\upsilon )\rho }}$$

Here $\upsilon$ is the Poisson ratio in the thickness direction. From Fig. 6, the elastic modulus of 〈110〉 and 〈100〉 crystal orientation is estimated to be approximately 264 GPa and 150 GPa, respectively, being in good agreement with the experimental data 237.6 GPa and 168.4 GPa^27^. The Poisson ratio in the thickness direction (z-direction) is calculated by the following equation:

11 $$\upsilon =\frac{1}{2}(-\frac{\partial {\varepsilon }_{x}}{\partial {\varepsilon }_{z}}-\frac{\partial {\varepsilon }_{y}}{\partial {\varepsilon }_{z}})$$

where *ε*_*x*_, *ε*_*y*_ and *ε*_*z*_ are the engineering strain in the *x*-, *y*- and *z*- directions, respectively.

Based on the above equation, the Poisson ratio are calculated, which is about 0.355 and 0.313 for the 〈100〉 and 〈110〉 crystal orientations, respectively. Here the density of copper is 8954 kg/m^3^ and assumed to be orientation-independent. As a results, the elastic wave speed for the 〈110〉 crystal orientation is about 6400 m/s, which is about 0.25 times higher than that (5200 m/s) in the 〈100〉 crystal orientation. From Fig. 5, the propagation speed of stress wave is estimated to be about 6600 m/s for 〈110〉 crystal orientation, which is about 35% higher than the value of its 〈100〉 counterpart. This discrepancy of elastic wave speed between theoretical prediction and the results of MD simulation can be attributed to the temperature-dependent elastic modulus and Poisson ratio together with the difference of mass density at the high temperature.

**Figure 7:**
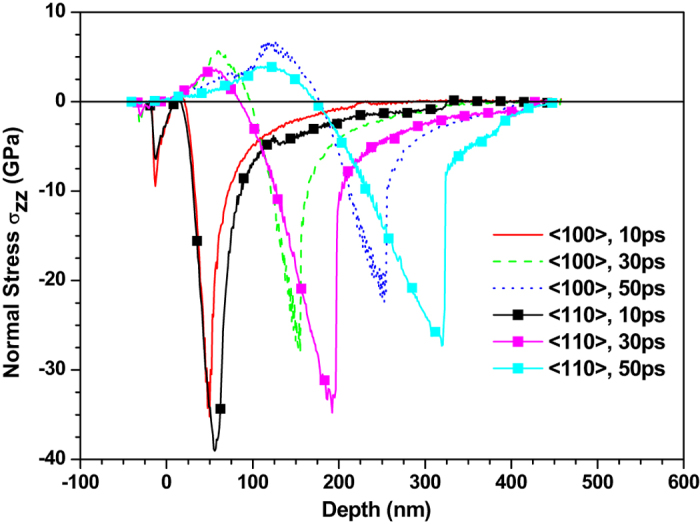
Comparison of normal stress (σ_zz_) distribution of 450 nm 〈100〉 and 〈110〉 copper films under an absorbed laser intensity of 0.065 J/cm^2^ at 10–50 ps time instant.

To show the effect of crystal orientation on the stress in thin film more accurately and clearly, we compare the stress in the 〈100〉 and 〈110〉 copper films in Fig. 7. The normal stress in the 〈110〉 copper film is found to be much higher than that in the 〈100〉 copper film. The distance between trough positions of normal stress wave in the 〈100〉 and 〈110〉 copper films is 48 nm at 30 ps and then increases to 90 nm at 50 ps, which is in good agreement with the difference (about 1600 m/s) between propagation speeds of the normal stress waves in these two crystal orientations. This proves again that the crystal orientation has significant effect on the stress of thin film.

**Figure 8:**
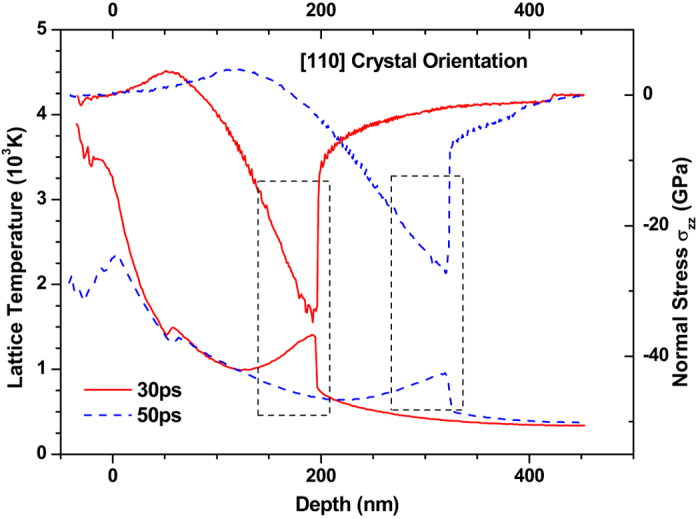
The contrast of spatial distributions of lattice temperature and normal stress (σ_zz_) in 450 nm 〈100〉 and 〈111〉 copper films under an absorbed laser intensity of 0.065 J/cm^2^ at 10–50 ps time instant.

An interesting phenomenon observed in the previous discussion is the bump in the curve of lattice temperature due to the thermo-mechanical coupling in the thin film. To present the coupling interaction between temperature and stress clearly, the spatial distributions of lattice temperature together with the normal stress in the 〈110〉 copper films are shown in Fig. 8. From Fig. 8, the trough position of normal stress wave is in coincidence with the position of bump of the lattice temperature, which agrees with the existing studies^8, 9^.

### Effect of Crystal Orientation on Femtosecond Laser-Induced Spallation Behaviors

**Figure 9:**
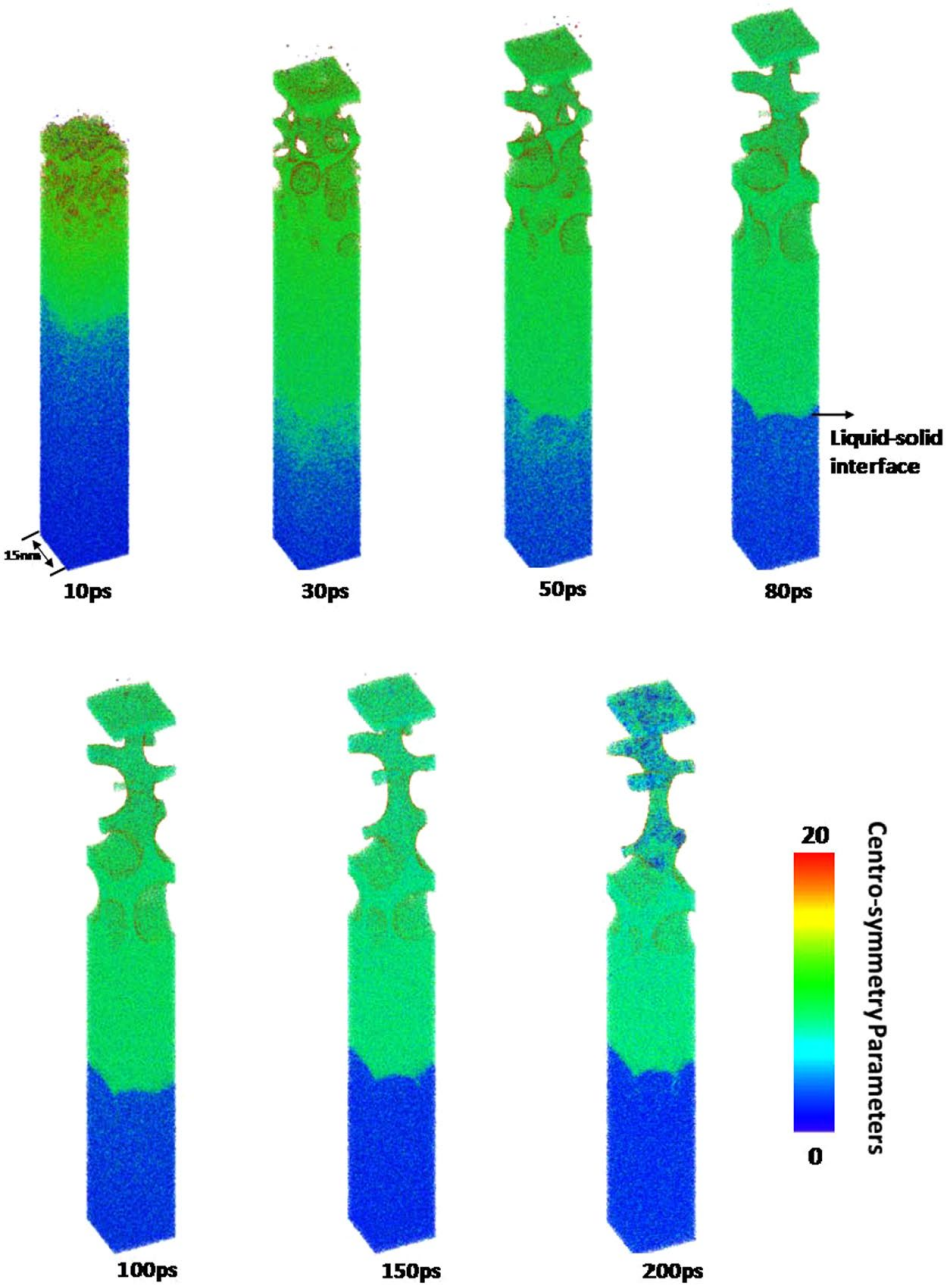
Snapshots of atomic configuration in the 0–100 nm part of 450 nm 〈100〉 copper film under an absorbed laser intensity of 0.065 J/cm^2^ at 10–200 ps time instant. The atoms are colored according to their centro-symmetry parameters.

**Figure 10:**
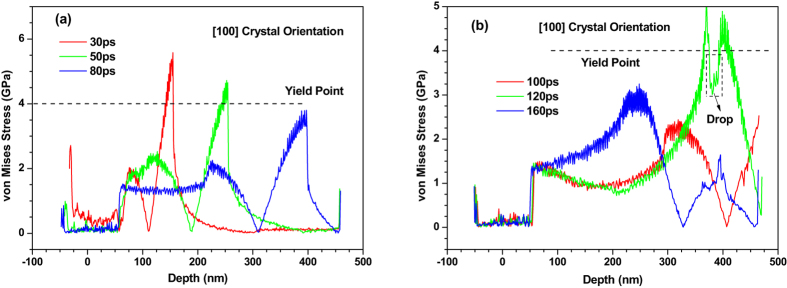
Equivalent stress (von Mises) distribution of 450 nm 〈100〉 copper films under an absorbed laser intensity of 0.065 J/cm^2^ at 30–160 ps time instant.

**Figure 11:**
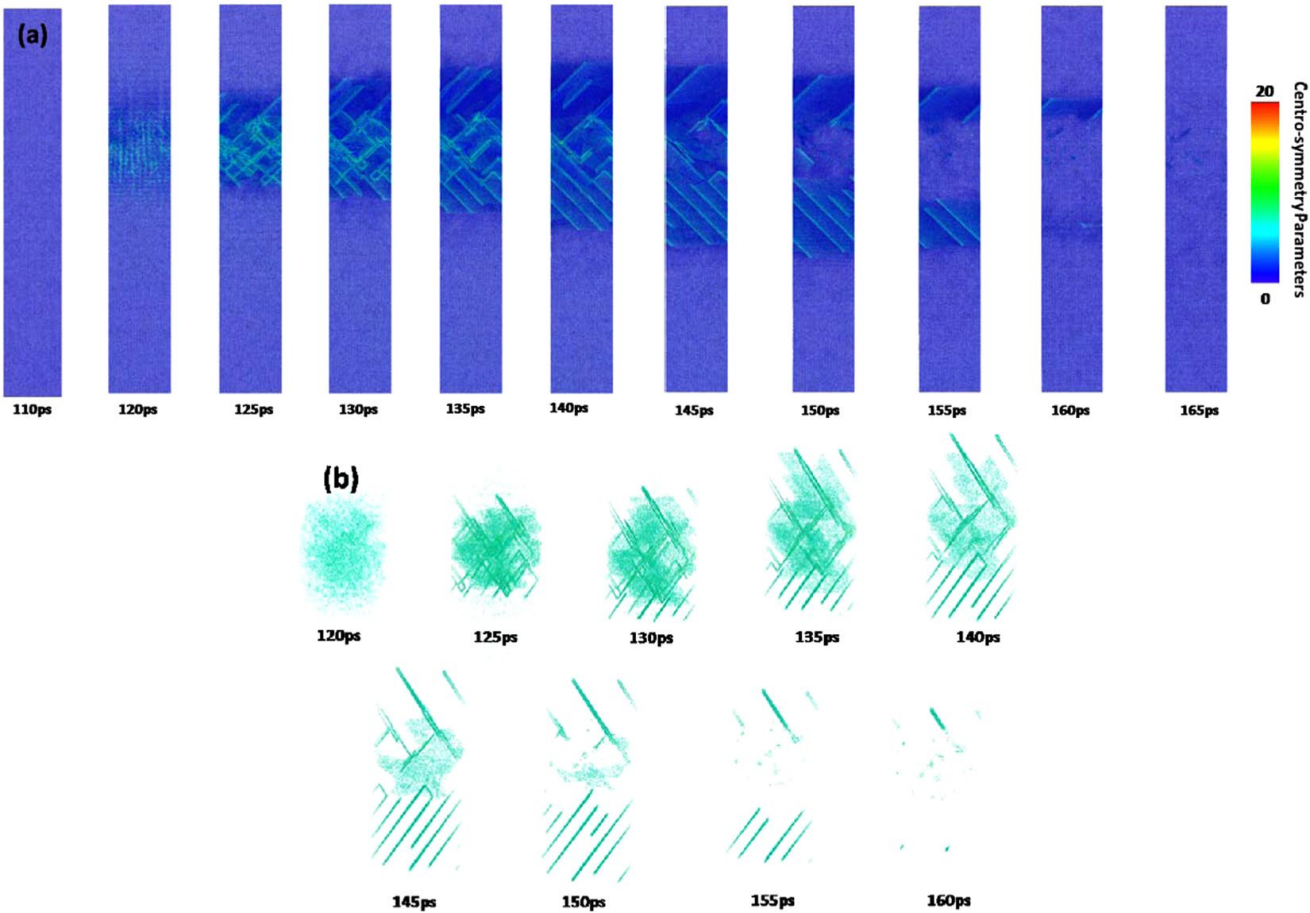
Snapshots of atomic configuration in the 350–450 nm part of 450 nm 〈100〉 copper film under an absorbed laser intensity of 0.065 J/cm^2^ at 110–165 ps time instant. The atoms are colored according to their centro-symmetry parameters, (**a**) 0 < CSP < 20, (**b**) CSP > 4.

**Figure 12:**
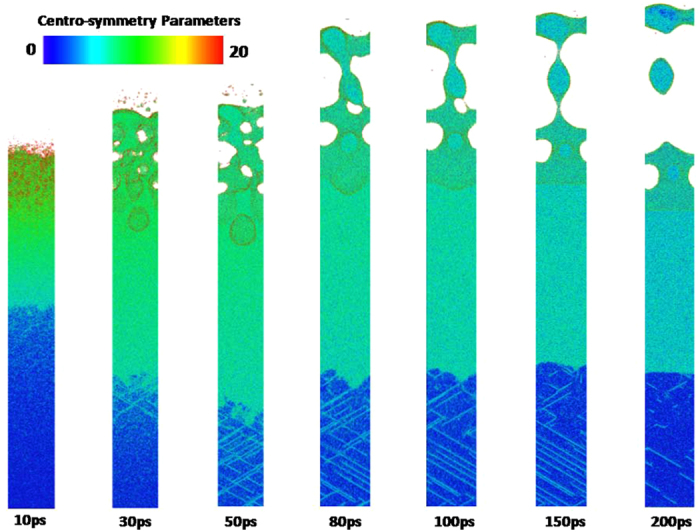
Snapshots of atomic centro-symmetry parameter in the 0–100 nm part of 450 nm 〈110〉 copper film under an absorbed laser intensity of 0.065 J/cm^2^ at 10–200 ps time instant. The atoms are colored according to their centro-symmetry parameters.

**Figure 13:**
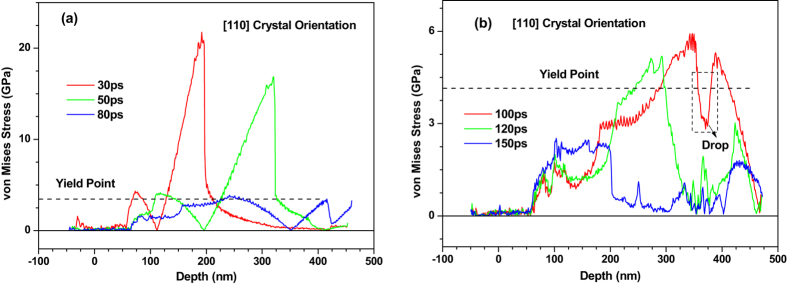
Equivalent stress (von Mises) distribution of 450 nm 〈110〉 copper films under an absorbed laser intensity of 0.065 J/cm^2^ at 30–150 ps time instant.

**Figure 14:**
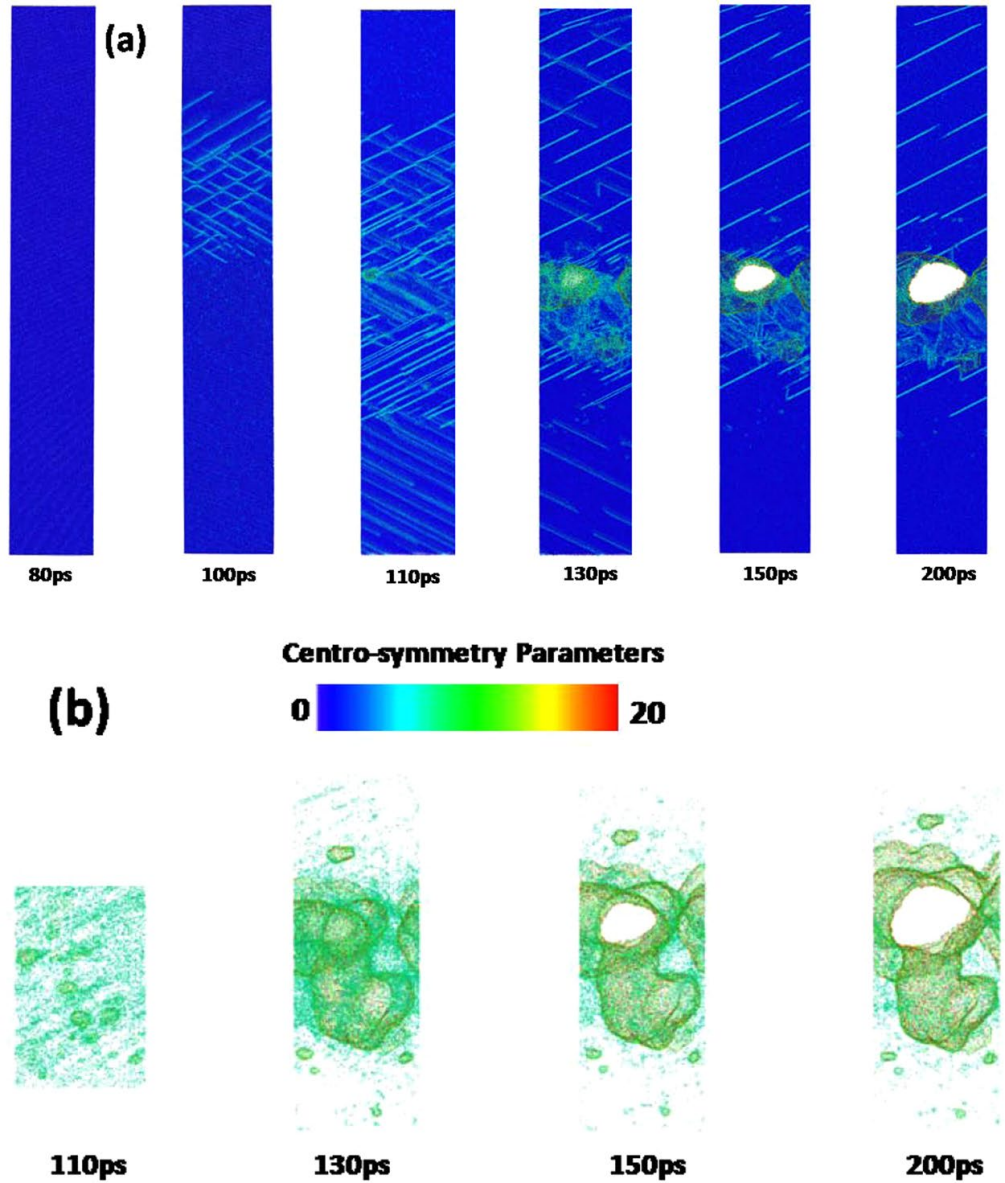
Snapshots of atomic centro-symmetry parameter in the 320–420 nm part of 450 nm 〈110〉 copper film under an absorbed laser intensity of 0.065 J/cm^2^ at 80–150 ps time instant. The atoms are colored according to their centro-symmetry parameters, (**a**) 0 < CSP < 20, (b) CSP > 4.

To demonstrate the effect of crystal orientation on the femtosecond laser-induced spallation behaviors of thin copper films, the snapshots of atomic configuration in the 〈100〉, 〈110〉 and 〈111〉 copper films under an absorbed laser intensity of 0.065 J/cm^2^ are provided in Figs 9–14. In these figures the atoms are colored according to their centro-symmetry parameters. The centro-symmetry parameter^28^ is calculated as follows:

12 $$CSP=\sum _{i=1}^{N/2}{|{\vec{R}}_{i}+{\vec{R}}_{i+N/2}|}^{2}$$

where ${\vec{R}}_{i}$ and ${\vec{R}}_{i+N/2}$ are the vectors from the central atom to a particular pair of nearest neighbors. The centro-symmetry parameter (CSP), which is a useful parameter to measure the local lattice disorder around an atom for solid-state systems, is used to determine whether the atom is a part of a perfect lattice or a local defect (e.g. a dislocation or stacking fault). For the atoms in a perfect lattice, the centro-symmetry parameter is zero. At a local defect the parameter is a larger positive value as the symmetry is broken. For example, the value of the centro-symmetry parameter is 0.5–4.0 for partial dislocations, while 4.0–20 for stacking faults.

It is worth pointing out that for the MD systems with smaller lateral dimensions (such as 5 nm × 5 nm), the spallation behaviors show discernible difference from that with larger lateral dimension (such as 15 nm × 15 nm used in the present study). However, when the lateral dimension is relatively large, the spallation behaviors seem be almost independent with the lateral dimension by making comparison between results of two cases of 15 nm × 15 nm and 45 nm × 45 nm. This is due to the fact that the laser-induced nanovoid interacts with itself across the periodic boundaries owing to smaller lateral dimension^29^. Thus, it is necessary to adopt a relatively large lateral dimension for investigating the femtosecond laser-induced spallation behavior of copper film.

The snapshots of 〈100〉 copper film shown in Fig. 9 illustrate femtosecond laser-induced atomic spallation behaviors near the irradiated surface. The irradiated surface region begins to melt after femtosecond laser irradiation. As a result, many nanovoids with diameter size of 0.1~1 nm are formed in the 0–20 nm part at 10 ps. As time goes on, the melting zone gradually spreads toward depth of film and the nanovoids rapidly gather to form larger voids whose sizes are more than 10 nm. An interesting phenomenon is that the maximum value of the melting depth is about 50 nm at 80 ps in Fig. 9, which is consistent with the prediction based on the lattice temperature shown in Fig. 2. Meanwhile, with the increase of time, the large voids gradually grow by means of aggregation of nanovoids.

In the whole femtosecond laser-induced process (0–0.2 ns), there is no any dislocation nucleation at the solid-liquid interface as shown in Fig. 9. For the three-dimensional stress state, the von Mises stress yield criterion^30^ can be used to reveal the mechanical mechanism of this phenomenon. The von Mises stress (equivalent stress) is given by

13 $${\sigma }_{Mises}=\sqrt{[{({\sigma }_{1}-{\sigma }_{2})}^{2}+{({\sigma }_{2}-{\sigma }_{3})}^{2}+{({\sigma }_{3}-{\sigma }_{1})}^{2}]}/\sqrt{2}$$

where *σ*_1_, *σ*_2_ and *σ*_3_ denotes *σ*_*zz*_, *σ*_*xx*_ and *σ*_*yy*_ respectively according to the stress state in the present work. Figure 10 shows equivalent stress (von Mises) distribution of 450 nm 〈100〉 copper films under an absorbed laser intensity of 0.065 J/cm^2^ at 30–160 ps. Before 80 ps the equivalent stress in most of the whole film is less than the yield stress of copper (about 3.5–4.0 GPa)^31^. Moreover, the region where the equivalent stress is larger than yield stress is still too small to form dislocation nucleation. These can be used to explain the phenomenon shown in Fig. 9.

It is worth noting that the stress cannot be released due to the fact that the dislocation dose not nucleate in thin film. The value of equivalent stress shown in Fig. 10 dose not vary dramatically from 30 ps to 80 ps. This result is quite different from the result that will be illustrated in the following 〈110〉 and 〈111〉 copper films.

After 100 ps, the equivalent stress in the non-irradiated surface region sharply increases due to the boundary reflection and reaches to yield stress of copper at 120 ps. In Fig. 11, the dislocation nucleates in the 350–450 nm part at 120 ps and a number of slip bands are formed accordingly. Meanwhile, a very interesting phenomenon occurs correspondingly, i.e., the equivalent stress of this region drops sharply due to dislocation nucleation (see Fig. 10).

However, the severe plastic deformation does not appear in the 350–450 nm part of 〈110〉 copper film. Therefore, when the equivalent stress decreases after 140 ps and is less than yield stress of copper as shown in Fig. 10, the slip band in the 350–400 nm part disappears gradually as shown in Fig. 11(a). From Fig. 11(b), the whole process of evolution of the slip band due to mechanical stress can be distinctly observed.

Figure 12 shows the snapshots of 〈110〉 copper film from 10 ps to 200 ps. All kinds of phenomena occurred in the 〈100〉 copper film is also observed in the 〈110〉 copper film. However, the spallation behaviors of the 〈100〉 and 〈110〉 copper films show significant differences. For instance, the maximum melting depth of 〈110〉 copper film reaches 60 nm which is 20% larger than that of 〈100〉 copper film. Moreover, the ablated surface region is found to quickly fly away from the 〈110〉 film substrate. In addition, dislocations nucleate and propagate at the liquid-solid interface at about 30 ps which form the slip band. The number of slip bands increases before 100 ps and then decreases. The equivalent stresses (von Mises) in the 〈110〉 copper film are shown in Fig. 13. The length of 〈110〉 copper film whose equivalent stress is larger than yield stress of copper can be up to 120 nm, which is sufficient to make dislocation nucleation and propagation. Thus, many slip bands are formed in the 〈110〉 copper film. However, the equivalent stresses (von Mises) in the 0–150 nm part of 〈110〉 copper film decease and become less than yield stress of copper after 100 ps which results in the decrease of the number of slip bands.

From the equivalent stresses (von Mises) in the 320–420nm part of 〈110〉 copper film shown in Fig. 13, dislocation nucleation and generation of slip band can be expected. The snapshots of atomic conformation in the 320–420 nm part of 450 nm 〈110〉 copper film under an absorbed laser intensity of 0.065 J/cm^2^ are presented in Fig. 14(a). As expected, many slip bands are formed and the large plastic deformation appears in this region, which further evolves into nano-cracks and void with the increase of time. These nano-cracks and void do not disappear with the decrease of equivalent stress in this region. From Fig. 14(b), it can be clearly observed that nano-cracks (or nano-voids) gradually grow as time goes on.

Compared with the equivalent stresses (von Mises) in the 〈100〉 copper film shown in Fig. 10, the equivalent stress (von Mises) in the 〈110〉 copper film falls sharply from 50 ps to 80 ps. The reason is that a large number of dislocation nucleation in this region makes the stress be released. Similarly, this also can explain why there is a sudden drop in the value of equivalent stress (von Mises) in the 〈110〉 copper film from 100 ps to 150 ps.

**Figure 15:**
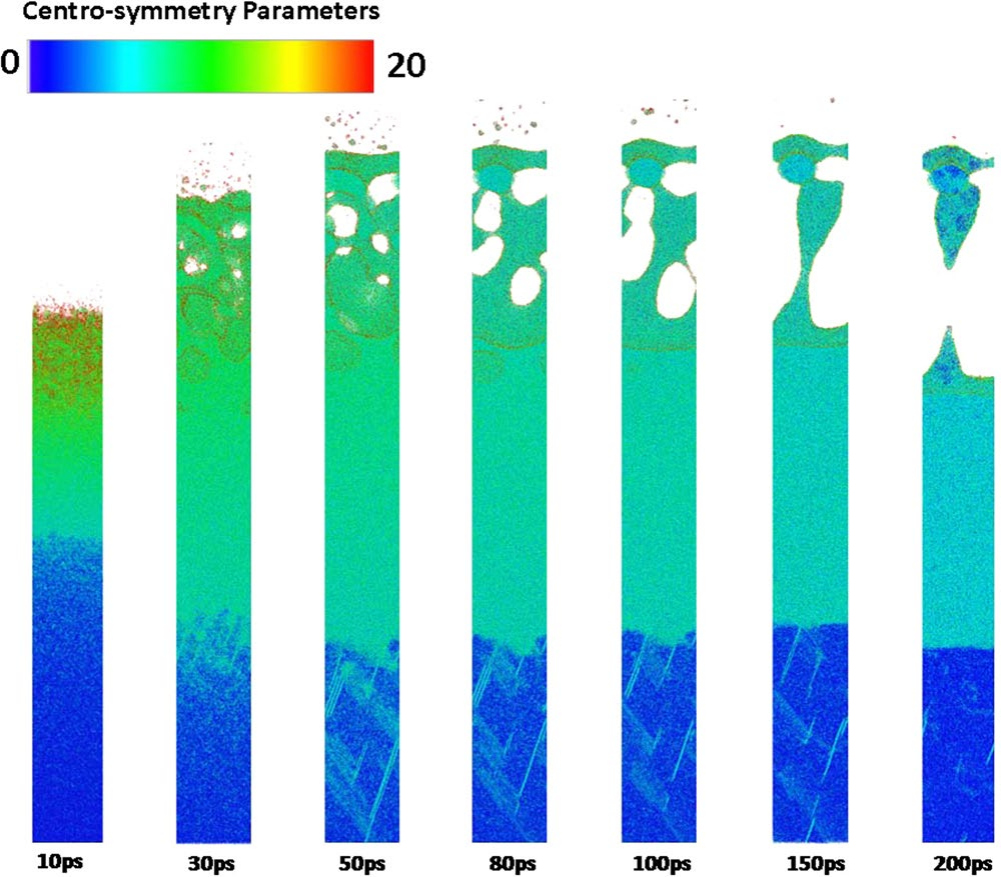
Snapshots of atomic centro-symmetry parameter in the 0–100 nm part of 450 nm 〈111〉 copper film under an absorbed laser intensity of 0.065 J/cm^2^ at 10–200 ps time instant.

**Figure 16:**
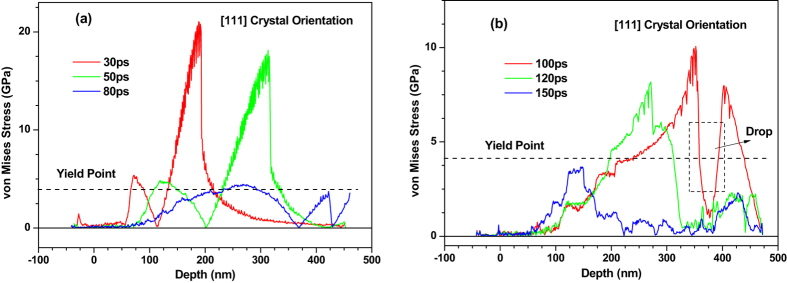
Equivalent stress (von Mises) distribution of 450 nm 〈111〉 copper films under an absorbed laser intensity of 0.065 J/cm^2^ at 30–150 ps time instant.

**Figure 17:**
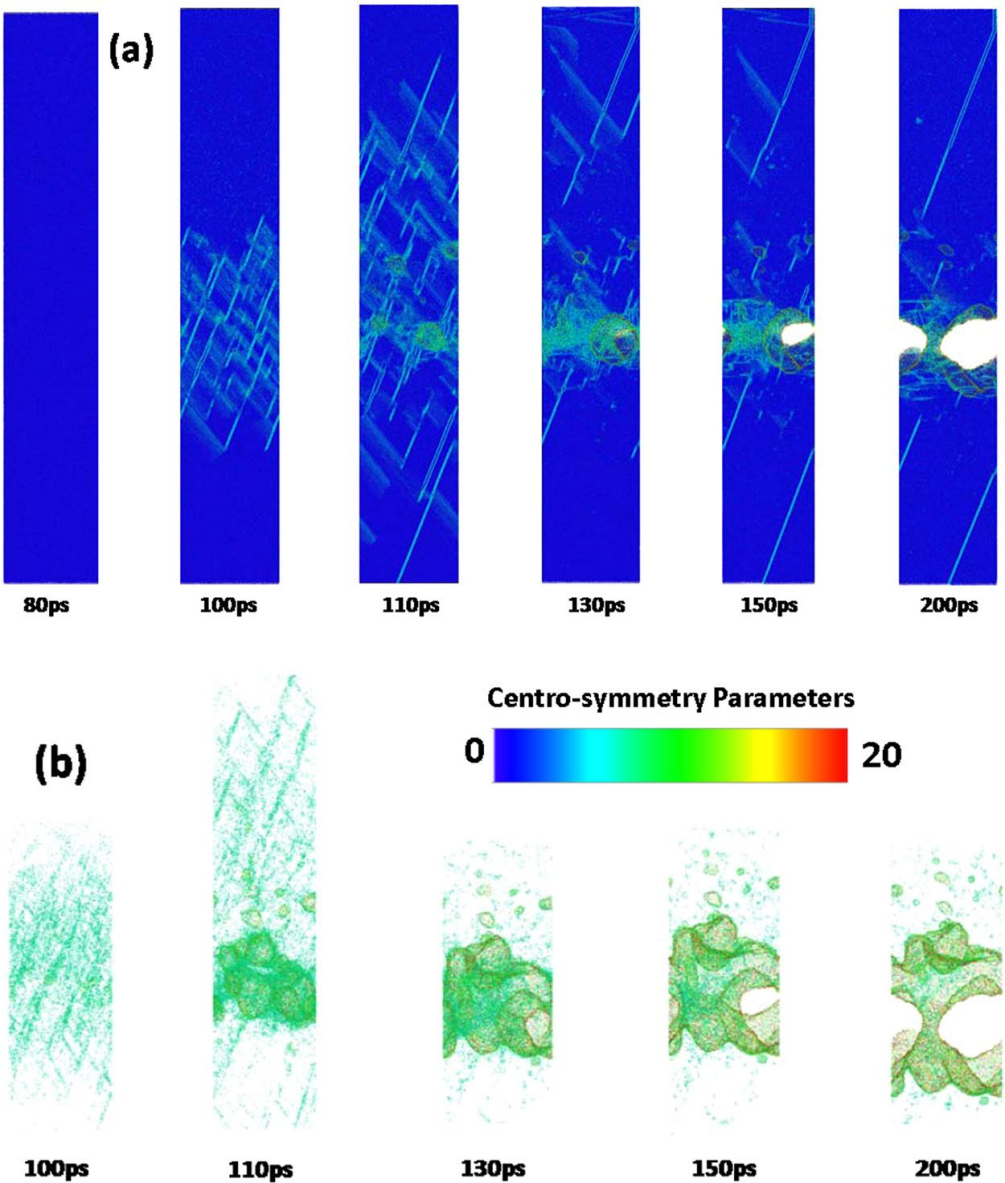
Snapshots of atomic centro-symmetry parameter in the 320–420 nm part of 450 nm 〈111〉 copper film under an absorbed laser intensity of 0.065 J/cm^2^ at 80–150 ps time instant. The atoms are colored according to their centro-symmetry parameters, (**a**) 0 < CSP < 20, (**b**) CSP > 4.

The snapshots of atomic configuration and the equivalent stresses (von Mises) in the 〈111〉 copper film are shown in Figs 15–17, respectively. The spallation behaviors occurred in the 〈111〉 copper film are very similar to that observed in the 〈110〉 copper film e.g., the maximum melting depth of 〈111〉 copper film is also about 60 nm, and the ablated surface region quickly flies away from the 〈111〉 film substrate. Dislocations nucleation and the slip bands occur at the liquid-solid interface to make the stress in the copper film be released. As a result, there is a distinct drop of the equivalent stresses (von Mises) in the 0–150 nm part of 〈111〉 copper film at 80 ps shown in Fig. 16.

However, the plastic deformation occurred in the 250–450 nm part of 〈111〉 copper film are more severe compared with that in the 〈110〉 copper film. As shown in Fig. 17, the fracture of the 〈111〉 copper film appears. Meanwhile, the equivalent stress (von Mises) in this region at 100 ps sharply falls as shown in Fig. 16.

Compared with the equivalent stresses (von Mises) in the 〈100〉 copper film shown in Fig. 10, the equivalent stress (von Mises) in the 〈111〉 copper film shown in Fig. 16 falls sharply from 50 ps to 80 ps within the 0–150 nm zone and from 100 ps to 150 ps within the 250–450 nm zone due to a large number of dislocation nucleation in this region.

## Conclusions

Monocrystal copper films with 〈100〉, 〈110〉 and 〈111〉 crystal orientations along the thickness direction are adopted to investigate the influence of crystal orientation on ultrafast thermo-mechanical responses and spallation behaviors based on MDS. The results are summarized as follows:

1. The crystal orientation has a significant influence on femtosecond laser-induced thermo-mechanical responses. Different crystal orientations lead to distinct differences in physical parameters of thin films, such as Young modulus, which further results in the difference of stress in films. Due to thermo-mechanical coupling interaction, discrepancy between normal stresses in copper films leads to the distinct differences in lattice temperature. For instance, the magnitude and propagation speed of normal stress wave in the 〈110〉 copper film are much higher than that in the 〈100〉 copper film.
2. The crystal orientation has a significant influence on femtosecond laser-induced spallation behaviors of copper films. The melting depth in the 〈100〉 copper film is 50 nm and the melting part dose not fly away from the shallow surface region, while the melting depth is 60 nm in the 〈110〉 and 〈111〉 copper films and the melting parts can fly away from the surface, which indicates that it is more difficult to ablate 〈100〉 copper film compared with 〈110〉 and 〈111〉 copper films. Moreover, the sizes of voids generated in melting subsurface in the 〈110〉 and 〈111〉 copper films are larger than that in the 〈100〉 copper film. The dislocation and slip band are formed and propagate from the solid-liquid interface in the 〈110〉 and 〈111〉 copper films, while these phenomena do not appear in the 〈100〉 copper film. In addition, many slip bands are generated in the non-irradiated surface region of copper films due to reflection of mechanical stress. These slip bands are developed into cracks (nanovoids), which further result in fracture of copper films.
